# Supplementary material for: COZOID: contact zone identifier for visual analysis of protein-protein interactions
Source: BMC Bioinformatics. 2018 Apr 6;19:125. doi: 10.1186/s12859-018-2113-6 (PMC5889581; doi:10.1186/s12859-018-2113-6)
Supplement: Supplementary file 2 — Software build. Executable binary file of the software tool. (ZIP 118,784 kb) [file 12859_2018_2113_MOESM2_ESM.zip › cozoid/java/docs/beansbinding-1.2.1-doc.zip!/org/jdesktop/observablecollections/package-summary.html]

org.jdesktop.observablecollections (Beans Binding)


---


|  |  |  |
| --- | --- | --- |
| **Overview** \| **Package** \| Class \| **Use** \| **Tree** \| **Deprecated** \| **Index** \| **Help** | |  |
| **PREV PACKAGE**   **NEXT PACKAGE** | **FRAMES**    **NO FRAMES**     **All Classes** |  |


---

## Package org.jdesktop.observablecollections

Provides support for observing changes to collection classes.

**See:**
  
          **Description**

| **Interface Summary** | |
| --- | --- |
| **ObservableList<E>** | A `List` that notifies listeners of changes. |
| **ObservableListListener** | Notification types from an `ObservableList`. |
| **ObservableMap<K,V>** | A `Map` that notifies listeners of changes to the `Map`. |
| **ObservableMapListener** | Notification types from an `ObservableMap`. |

| **Class Summary** | |
| --- | --- |
| **ObservableCollections** | `ObservableCollections` provides factory methods for creating observable lists and maps. |
| **ObservableCollections.ObservableListHelper<E>** | `ObservableListHelper` is created by `observableListHelper`, and useful when changes to individual elements of the list can be tracked. |

## Package org.jdesktop.observablecollections Description

Provides support for observing changes to collection classes.
The classes in this package are not part of JSR 295.

---


|  |  |  |
| --- | --- | --- |
| **Overview** \| **Package** \| Class \| **Use** \| **Tree** \| **Deprecated** \| **Index** \| **Help** | |  |
| **PREV PACKAGE**   **NEXT PACKAGE** | **FRAMES**    **NO FRAMES**     **All Classes** |  |


---
